# Prevalence and risk factors for metabolic dysfunction–associated steatotic liver disease in Sweden: Insights from the SCAPIS cohort

**DOI:** 10.1111/joim.70071

**Published:** 2026-02-05

**Authors:** Oumarou Nabi, Jonas Spaak, Göran Bergström, Gunnar Engström, Carl Johan Östgren, Andrei Malinovschi, Joel Kullberg, Anders Blomberg, Tomas Jernberg, Daniel P. Andersson, Hannes Hagström

**Affiliations:** ^1^ Department of Medicine Huddinge, Karolinska Institutet Stockholm Sweden; ^2^ Department of Clinical Sciences Danderyd Hospital Karolinska Institutet Stockholm Sweden; ^3^ Department of Molecular and Clinical Medicine University of Gothenburg Sahlgrenska Academy Gothenburg Sweden; ^4^ Department of Clinical Physiology Västra Götalandsregionen Gothenburg Sweden; ^5^ Department of Clinical Science in Malmö Lund University Lund Sweden; ^6^ Department of Health Medicine and Caring and Sciences Linköping University Linköping Sweden; ^7^ Centre of Medical Image Science and Visualization (CMIV) Linköping University Linköping Sweden; ^8^ Department of Medical Sciences Clinical Physiology Uppsala University Uppsala Sweden; ^9^ Department of Surgical Sciences, Radiology, Uppsala University Uppsala Sweden; ^10^ Antaros Medical AB Mölndal Sweden; ^11^ Department of Public Health and Clinical Medicine Umeå University Umeå Sweden; ^12^ Department of Endocrinology C2:94 Karolinska University Hospital Huddinge Stockholm Sweden; ^13^ Division of Hepatology Department of Upper GI Diseases Karolinska University Hospital Stockholm Sweden

**Keywords:** advanced fibrosis, MASLD, prevalence

## Abstract

**Background and Aims:**

Metabolic dysfunction–associated steatotic liver disease (MASLD) is the most common chronic liver disease globally, but its prevalence and severity remain poorly characterized in the general population. Our aim was to estimate the prevalence of MASLD and the risk of advanced fibrosis in a large Swedish general population cohort.

**Methods:**

From the Swedish CArdioPulmonary bioImage Study (SCAPIS) cohort, we analyzed 27,763 participants aged 50–64 years who underwent extensive clinical characterization. MASLD was defined as <48 HU on non‐contrast liver computed tomography (CT) imaging. The risk for advanced fibrosis was assessed using the dynamic aspartate aminotransferase (AST)/alanine transaminase (ALT) ratio.

**Results:**

MASLD was present in 18.1% of participants and was more common in men than women (25.5% vs. 11.2%). Prevalence increased with cardiometabolic burden: from 7.0% among those without obesity, hypertension, or Type 2 diabetes mellitus (T2DM) to 70.2% among those with all three conditions. MASLD risk was elevated in individuals with obesity alone (adjusted odds ratio [aOR] 5.56; 95% CI = 4.89–6.31), T2DM alone (aOR = 2.66; 95% CI = 2.13–3.33), or hypertension alone (aOR = 1.78; 95% CI = 1.59–1.99). The combination of all three conferred the highest risk (aOR = 17.1; 95% CI = 14.0–20.9). Among persons with MASLD, 24.8% were classified as at risk for advanced fibrosis. Fibrosis risk was independently associated with hypertension (aOR = 1.44; 95% CI = 1.24–1.66), T2DM (aOR = 1.24; 95% CI = 1.06–1.46), male sex (aOR = 1.20; 95% CI = 1.02–1.42), and alcohol consumption (aOR per gram/day = 1.02; 95% CI = 1.01–1.03).

**Conclusions:**

In Sweden, almost one in five middle‐aged adults is affected by MASLD, with a quarter of cases at risk of advanced fibrosis. Male sex, obesity, T2DM, and hypertension are important predictors of the prevalence and severity of MASLD.

## Graphical abstract

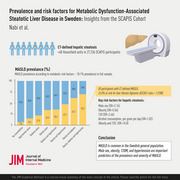

**Abbreviations**
AASLD: Association for the Study of Liver Diseases
AIC: Akaike information criterion
ALT: alanine transaminase
aOR: adjusted odds ratios
ARR: AST/ALT ratio
AST: aspartate aminotransferase
BMI: body mass index
CI: confidence intervals
CKD: chronic kidney disease
CT: computed tomography
CVD: cardiovascular disease
dAAR: dynamic aspartate‐to‐alanine aminotransferase ratio
DBP: diastolic blood pressure
EASL: European Association for the Study of the Liver
FLI: fatty liver index
GGT: gamma‐glutamyl transferase
HbA1c: hemoglobin A1C
HBP: high blood pressure
HCC: hepatocellular carcinoma
HDL‐C: high‐density lipoprotein cholesterol
HU: Hounsfield units
ICD: International Classification of Diseases
IFG: impaired fasting glucose
LDL‐C: low‐density lipoprotein cholesterol
MASLD: metabolic dysfunction–associated steatotic liver disease
MRI: magnetic resonance imaging
NAFLD: nonalcoholic fatty liver disease
SBP: systolic blood pressure
SCAPIS: Swedish CArdioPulmonary bioImage Study
SD: standard deviations
SLD: steatotic liver disease
T2DM: Type 2 diabetes mellitus
U/L: units per liter
VIFs: variance inflation factors

## 1 Introduction

Metabolic dysfunction–associated steatotic liver disease (MASLD), formerly known as nonalcoholic fatty liver disease (NAFLD), is closely linked to obesity and Type 2 diabetes mellitus (T2DM) and is the most prevalent chronic liver disease globally, affecting an estimated 38% of adults worldwide [1]. MASLD is defined by hepatic steatosis in the presence of at least one metabolic risk factor, such as obesity, T2DM, hypertension, or dyslipidemia [2].

Despite having a high prevalence, MASLD remains substantially under‐recognized, especially in primary care settings, where early detection before progression to cirrhosis is most attractive [3, 4, 5, 6, 7, 8]. Barriers to timely diagnosis include limited clinician awareness and low uptake of screening guidelines [3, 4, 5, 6, 7, 8].

In contrast to its growing burden, robust population‐level data on MASLD are scarce in the Nordic countries, where rates of obesity and T2DM are generally lower than in other high‐income countries. Existing prevalence estimates are often derived from hospital‐based or biopsy‐confirmed cohorts or often rely on small sample sizes that limit generalizability [9, 10, 11]. Furthermore, the sociodemographic and metabolic distribution of MASLD remains poorly characterized, hindering the design of effective, equity‐focused screening and prevention strategies. Identifying high‐risk subgroups can be informative to improve early diagnosis of hepatic fibrosis due to MASLD and reduce long‐term complications, including cirrhosis and hepatocellular carcinoma. This may be especially important now when approved pharmacotherapies exist and several more are anticipated to be available in the coming years.

Here, we analyzed data from the Swedish CArdioPulmonary bioImage Study (SCAPIS), a large, multicenter, population‐based study of middle‐aged (50–64‐year‐old) adults [12]. Using standardized non‐contrast‐enhanced abdominal computed tomography (CT) to quantify liver steatosis, we (1) estimated the prevalence of MASLD in Sweden, (2) characterized its distribution across sociodemographic and cardiometabolic subgroups, and (3) identified independent predictors.

## 2 Materials and methods

### 2.1 Study population

This cross‐sectional study utilized baseline data from SCAPIS, a population‐based study comprising 30,154 adults aged 50–64 years, recruited between 2013 and 2018 at six Swedish university hospitals (Gothenburg, Linköping, Malmö/Lund, Stockholm, Umeå, and Uppsala) [12]. Participants were randomly selected from the national population registry and stratified by sex and study site to ensure demographic and regional representativeness. At each study center, participants underwent standardized physical examinations, anthropometric measurements, and fasting blood sampling. They also completed a comprehensive questionnaire covering sociodemographic characteristics, lifestyle behaviors, and medical history. A screening questionnaire was also administered to identify contraindications for CT imaging. Of the eligible participants, approximately 97% underwent non‐contrast abdominal CT, with a small proportion (903 individuals) declining due to personal preferences. A detailed description of the SCAPIS protocol has been published previously [12]. Here, we further excluded individuals with excessive alcohol intake (≥30 g/day for men and ≥20 g/day for women) or a prior diagnosis of chronic liver disease except MASLD and diagnoses related to alcohol overconsumption. Disease history was ascertained through linkage with Swedish national inpatient and outpatient health registers up to 2018 using harmonized International Classification of Diseases (ICD‐8, ICD‐9, and ICD‐10) codes (Table S1). Excluded conditions included alcohol‐related liver disease (e.g., alcoholic steatosis, hepatitis, or cirrhosis), chronic viral hepatitis (B or C), autoimmune liver diseases (e.g., autoimmune hepatitis, primary biliary cholangitis, and primary sclerosing cholangitis), genetic liver disorders (e.g., Wilson disease, hemochromatosis, and alpha‐1 antitrypsin deficiency), non‐autoimmune cholestatic liver diseases, vascular liver conditions (e.g., Budd–Chiari syndrome), and cryptogenic cirrhosis without metabolic dysfunction. Participants without suitable non‐contrast abdominal CT imaging for liver fat quantification were also excluded.

### 2.2 Definition of metabolic dysfunction–associated steatotic liver disease (MASLD)

We defined MASLD based on the presence of liver fat and at least one indicator of metabolic dysfunction and no excess alcohol consumption, in accordance with the definition [2]. Hepatic steatosis was identified by a liver attenuation value of <48 HU on non‐contrast CT, a threshold widely used in the literature to indicate moderate to severe liver fat infiltration. Although various HU cutoffs (e.g., 57, 48, 40, and 37) have been proposed [13, 14, 15], the threshold of 48 HU was chosen as it is the most consistently applied and validated in epidemiologic and clinical studies for diagnosing clinically relevant steatosis. A comprehensive description of the CT imaging protocol employed in the SCAPIS study is provided in a prior publication [16]. Briefly, liver attenuation was quantified using an automated approach from a single‐slice CT image. The automated segmentations were performed using convolutional neural networks with UNet‐based architectures. The networks were trained using reference segmentations of large numbers of slices (*n* = 2681) acquired from a single center. The reference segmentations were created using a semiautomated approach.

Automated segmentation methodology previously described [16] was used to generate segmentation proposals. These proposals were corrected and updated using trained image analysts and a radiologist at Antaros Medical. The automated segmentations were evaluated using eightfold cross validation and showed strong agreement with the reference segmentations: 0.971 Dice, 2.69% mean average percent error. All segmentation results were visually quality controlled. The readout of the final measurements included areas as well as mean and median attenuation (in HU) of each individual tissue target.

Additional readouts of mean attenuation values robust to outliers (*r*_mean_) were performed for liver and muscle attenuation readouts. In these, a filtering of mean ± 2.5 standard deviations (SD) was used to exclude pixel‐wise outlier image intensities.

Metabolic dysfunction was defined in the steatotic liver disease nomenclature definition as at least one of the following: (1) overweight or obesity, defined as a body mass index (BMI) ≥25 kg/m^2^; (2) T2DM, based on self‐reported diagnosis, current use of glucose‐lowering medication, fasting plasma glucose ≥7.0 mmol/L, or elevated hemoglobin A1C (HbA1C > 6.4%); (3) prediabetes, defined by fasting plasma glucose between 5.6 and 6.9 mmol/L or HbA1c between 5.7% and 6.4%; (4) prehypertension, defined as systolic blood pressure (SBP) ≥135 mmHg, diastolic blood pressure (DBP) ≥85 mmHg, or use of antihypertensive medication; (5) low high‐density lipoprotein cholesterol (HDL‐C), defined as <1.0 mmol/L in men (<1.3 mmol/L in women); or (6) hypertriglyceridemia, defined as triglyceride levels ≥1.7 mmol/L or use of lipid‐lowering medication. All blood biomarkers from the SCAPIS dataset that were not originally reported in international units were converted to their corresponding international units for consistency and comparability.

### 2.3 Estimation of liver fibrosis

As the SCAPIS cohort was primarily focused on cardiovascular and pulmonary risk assessment, liver biopsy, imaging‐based liver fibrosis assessment, and routine laboratory parameters for liver fibrosis estimation such as platelet count were unavailable. This precluded us from using commonly recommended noninvasive fibrosis scores such as FIB‐4. Given this, liver fibrosis was estimated using the dynamic aspartate‐to‐alanine aminotransferase ratio (dAAR), a validated, noninvasive biochemical index that incorporates serum aspartate aminotransferase (AST), alanine transaminase (ALT), and age to predict the risk of advanced liver fibrosis and future severe liver‐related outcomes [17]. Here, the dAAR was selected as a pragmatic and reproducible surrogate marker for fibrosis risk stratification in population‐based settings. The dAAR was computed in accordance with the published formula [17]. Using a cutoff of 1.5708, this approach enables reasonably accurate classification of advanced fibrosis, with a sensitivity of 0.71 and specificity of 0.75, in the absence of imaging or biopsy‐based diagnostics.

### 2.4 Covariates

Sociodemographic, clinical, lifestyle, and biochemical covariates were assessed using standardized protocols from the SCAPIS study, including self‐administered questionnaires, physical examinations, and fasting blood samples analyzed in certified laboratories [12]. Sociodemographic variables comprised age and sex. Educational attainment was categorized into three levels. Primary education included individuals who either did not complete primary school or completed only primary school or its equivalent, typically up to 9 years of schooling. Secondary education encompassed those who completed high school, vocational training, folk high school, or other equivalent forms of education, usually totaling up to 12 years of schooling. Tertiary education (or higher education) referred to individuals who had obtained a degree from a university or college, corresponding to more than 12 years of schooling. Geographic origin was defined based on the birthplace of participants and both parents and categorized as Swedish, other European, African, Asian, South American, or North American. Participants were considered of foreign origin if they or both parents were born outside Sweden. Anthropometric data included height, weight, and waist and hip circumferences; BMI was categorized as normal (<25 kg/m^2^), overweight (25.0–29.9 kg/m^2^), and obese (≥30.0 kg/m^2^), with lower thresholds for Asian participants (overweight: 23.0–24.9 kg/m^2^; obesity: ≥25.0 kg/m^2^). Hypertension (high blood pressure) was defined as SBP ≥140 mmHg and/or DBP ≥90 mmHg, or use of antihypertensive medications, and further classified into five categories: no hypertension, prehypertension (SBP 130–139 mmHg or DBP 85–89 mmHg), untreated hypertension, treated and controlled hypertension (SBP <140 mmHg and DBP <90 mmHg), or treated but uncontrolled hypertension. Diabetes status included normoglycemia (fasting glucose <5.6 mmol/L and no antidiabetic treatment), impaired fasting glucose (IFG)/prediabetes (5.6–6.9 mmol/L), newly diagnosed (previously unrecognized diabetes based on elevated fasting glucose at the time of study inclusion), known, treated, and controlled diabetes, and known diabetes treated but uncontrolled based on an HbA1c >6.4%. Hyperlipidemia was defined as hypercholesterolemia (total cholesterol) >5.5 mmol/L, HDL‐C <1.0 mmol/L for men (<1.3 mmol/L for women), triglycerides ≥1.7 mmol/L, or lipid‐lowering medication use and further categorized into treated and untreated forms. Participants were grouped into mutually exclusive metabolic comorbidity profiles: no metabolic condition, isolated obesity, isolated diabetes, isolated hypertension, or combinations of these. Lifestyle factors included smoking status (never, former, and current), frequencies of coffee and sugar‐sweetened beverage (soda) consumption, categorized from ≤2 times per week to ≥5 times per day, and physical activity level (sedentary, moderate but not regular, moderate with regularity, and regular). Sedentary activity includes individuals who spend most of their free time in low‐energy behaviors, such as reading, watching television, or using a computer, and engage in walking, cycling, or other physical activity for less than 2 h per week. Moderate activity, irregular, refers to those who walk, cycle, or move in other ways for at least 4 h per week, but not as part of a structured routine. This includes activities such as walking or cycling to work, casual walks, heavier housework, gardening, fishing, table tennis, or bowling. Moderate and regular activity describes individuals who participate in more structured activities such as running, swimming, tennis, badminton, gymnastics, or similar, typically 1–2 times per week for at least 30 min per session, totaling an average of 2–3 h per week. Regular intense exercise includes individuals who engage in frequent, vigorous physical activity such as training or competing in sports like running, orienteering, skiing, swimming, football, or handball, or regularly performing sports like tennis, badminton, or gymnastics at least three times per week, with each session lasting at least 30 min. Alcohol intake was treated as a continuous variable.

### 2.5 Statistical analysis

Baseline characteristics were summarized as means with SD for continuous variables and frequencies with percentages for categorical variables. Participants were stratified into MASLD and non‐MASLD groups, with descriptive statistics presented for the overall sample and by MASLD status. Variables with missing data in fewer than 3% of cases were analyzed using complete‐case analysis; no imputation was applied.

MASLD prevalence and associated characteristics were estimated using exact binomial 95% confidence intervals (CIs). Subgroup analyses were conducted by sex, geographic origin, education level, smoking status, BMI category, T2DM status (no T2DM, IFG, newly diagnosed T2DM, treated T2DM, and elevated HbA1c), hypertension status (normotensive, prehypertensive, untreated, treated‐controlled, and treated‐uncontrolled), and dyslipidemia status (none, untreated, and treated). Cardiometabolic multimorbidity (obesity, T2DM, and hypertension) was assessed using both individual and mutually exclusive combinations of obesity, T2DM, and hypertension, selected for their clinical importance and prevalence, ensuring statistical power and interpretability.

Associations between MASLD and potential determinants were assessed using univariable and multivariable logistic regression models. Explored covariates included age, sex, BMI, waist circumference, T2DM, hypertension, components of dyslipidemia, liver enzymes (AST, ALT, and GGT), triglycerides, total cholesterol, HDL‐C, and behavioral factors (physical activity, alcohol intake, smoking status, and coffee and soda consumption). Three main models examined the influence of metabolic comorbidities (obesity, T2DM, and hypertension): Model 1 treated obesity, T2DM, and hypertension as binary indicators; Model 2 incorporated categorical definitions of each condition; and Model 3 included mutually exclusive multimorbidity (obesity, T2DM, and hypertension) profiles.

Model 3 included all covariates from Model 1, with additional adjustment for waist circumference. To account for potential interaction and collinearity among comorbid conditions, the individual variables for obesity, T2DM, and hypertension were replaced with a mutually exclusive composite variable capturing all eight possible combinations of these conditions (Model 3). This approach allowed for a more nuanced assessment of their joint contribution to advanced liver fibrosis risk.

Liver fibrosis was analyzed in the subpopulation of MASLD using univariable and two multivariable logistic regression models. Model 1 included sociodemographic characteristics (sex, geographic origin, and education), individual metabolic comorbidities (obesity, T2DM, and hypertension), dyslipidemia, and lifestyle factors (physical activity, alcohol intake, smoking, and coffee and soda consumption), representing a parsimonious baseline model. Model 2 added waist circumference, triglycerides, and cholesterol and treated comorbidities (obesity, T2DM, and hypertension) as a combined categorical multimorbidity variable to evaluate whether more complex clustering of risk factors modified the observed associations. Given that age, ALT, and AST are integral components of the dAAR score, they were excluded from the liver fibrosis models to prevent multicollinearity and ensure model integrity.

All models included an interaction term between alcohol and tobacco and were selected using bidirectional stepwise regression based on the Akaike information criterion (AIC) to balance model fit and parsimony. Model diagnostics included variance inflation factors (VIFs) to assess multicollinearity. Sensitivity analyses were performed to test the robustness of model estimates. Models, including BMI without waist circumference consistently, had superior fit (lower AIC) and were retained over alternatives, including waist circumference alone. Adjusted odds ratios (aORs) and 95% CIs were reported for all final models. All analyses were conducted using R software (version 4.4.2). Statistical tests were two‐sided, with significance defined as *p* < 0.05. To control multiple testing, *p*‐values were adjusted using the Bonferroni method, applied to subgroup comparisons and model‐based inference as appropriate.

## 3 Results

### 3.1 Study population

**Fig. 1:**
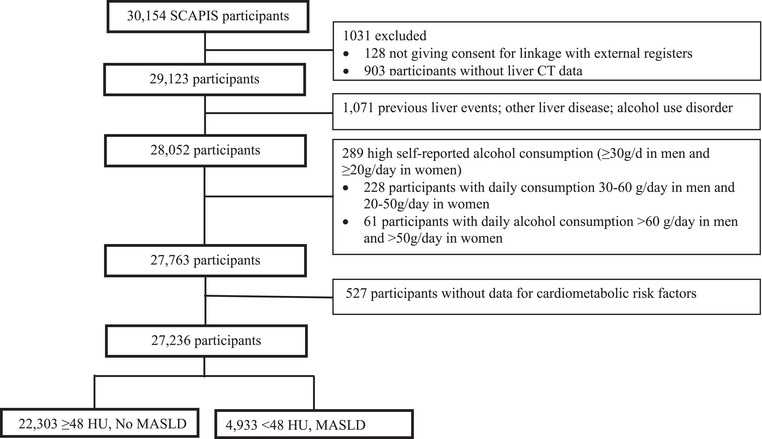
Study population flow chart.

At the time of analysis, data were available for 30,154 participants. Of these, 128 individuals were excluded due to lack of consent for registry linkage. Additionally, 1071 individuals with a history of liver disease and 903 individuals without available CT imaging data were excluded. Among the remaining 28,052 participants, 228 (0.81%) individuals met criteria for MetALD (liver attenuation <48 HU and daily alcohol consumption of 30–60 g in men and 20–50 g/day in women), and 61 (0.22%) individuals reported alcohol consumption >60 g/day for men or >50 g/day in women; both groups were subsequently excluded from the analysis. After exclusions, 27,763 participants were retained for the final analysis (Fig. 1). Baseline characteristics of the overall cohort, stratified by MASLD status, are presented in Table 1.

**Table 1:** General population characteristics of the study population according to the presence of metabolic dysfunction–associated steatotic liver disease (MASLD)

Characteristics^a^	All population *N* = 27,236	No MASLD *N* = 22,303	MASLD *N* = 4933
Age, year	57.5 (4.3)	57.4 (4.3)	58.1 (4.3)
Sex			
Women	14,066 (51.6%)	12,497 (56.0%)	1569 (31.8%)
Men	13,170 (48.4%)	9806 (44.0%)	3364 (68.2%)
Geographic origin			
Swedish	22,376 (82.2%)	18,451 (82.7%)	3925 (79.6%)
Europe	3354 (12.3%)	2650 (11.9%)	704 (14.3%)
North America	66 (0.2%)	53 (0.2%)	13 (0.3%)
South America	255 (0.9%)	192 (0.9%)	63 (1.3%)
Asia	1,035 (3.8%)	824 (3.7%)	211 (4.3%)
Africa	150 (0.6%)	133 (0.6%)	17 (0.3%)
Education level			
Primary education	2355 (8.7%)	1749 (8.0%)	606 (12.7%)
Secondary education	12,093 (45.5%)	9605 (44.1%)	2488 (52.1%)
Tertiary education	12,122 (45.6%)	10,444 (47.9%)	1678 (35.2%)
Height, cm	172.3 (9.7)	171.8 (9.6)	174.7 (9.7)
Weight, kg	80.2 (15.8)	77.0 (13.8)	94.8 (16.3)
Waist circumference, cm	94.2 (12.9)	91.3 (11.3)	107.2 (11.3)
Hip circumference, cm	102.6 (8.8)	101.3 (8.1)	108.2 (9.9)
BMI, kg/m^2^	27.0 (4.4)	26.1 (3.8)	31.0 (4.7)
BMI class			
Normal weight	9646 (35.8%)	9371 (42.5%)	275 (5.6%)
Overweight	11,544 (42.8%)	9567 (43.4%)	1977 (40.2%)
Obese	5758 (21.4%)	3097 (14.1%)	2661 (54.2%)
Systolic blood pressure, mmHg	125.7 (16.9)	124.1 (16.7)	133.0 (16.0)
Diastolic blood pressure, mmHg	77.4 (10.5)	76.5 (10.4)	81.9 (9.9)
High blood pressure, yes	9374 (34.4%)	6632 (29.7%)	2742 (55.6%)
Hypertension treatment, yes	5245 (19.3%)	3580 (16.1%)	1665 (33.8%)
T2DM, yes	2653 (9.7%)	1444 (6.5%)	1209 (25.0%)
Known T2DM	1252 (4.6%)	632 (2.8%)	620 (12.6%)
Antidiabetic drug use (% of known T2DM)	959 (76.6%)	483 (76.4%)	474 (76.5%)
Glucose, mmol/L	5.8 (1.1)	5.6 (0.9)	6.5 (1.7)
HbA1c, %	5.5 (0.6)	5.4 (0.4)	5.7 (0.8)
Insulin, mIU/L	7 (18.7)	6 (8.9)	13 (39.2)
ALT, U/L	27 (17)	24 (13)	40 (23)
AST, U/L	26.2 (11.0)	25.2 (9.6)	30.8 (15.2)
GGT, IU/L	31.8 (40.7)	27.6 (29.5)	50.8 (69.0)
Total cholesterol, mmol/L	5.5 (1.1)	5.5 (1.0)	5.4 (1.1)
LDL‐C, mmol/L	3.5 (1.0)	3.45 (1.0)	3.5 (1.0)
HDL‐C, mmol/L	1.6 (0.5)	1.7 (0.5)	1.3 (0.4)
TGs, mmol/L	1.2 (0.8)	1.1 (0.6)	1.8 (1.3)
Hyperlipidemia, yes	9592 (35%)	6911 (31%)	2681 (5%)
Creatinine, mmol/L	77.1 (16.2)	77.4 (16.2)	79.9 (16.1)
eGFR CKD EPI mL/min/m^2^	88.7 (12.2)	88.4 (12.1)	90 (12.3)
Smoking status			
Never	13,685 (51.8%)	11,564 (53.4%)	2121 (44.5%)
Former smoker	9572 (36.2%)	7606 (35.1%)	1966 (41.3%)
Current	3168 (12.0%)	2492 (11.5%)	676 (14.2%)

Abbreviations: ALT, alanine transaminase; AST, aspartate aminotransferase; BMI, body mass index; eGFR CKD EPI, estimated glomerular filtration rate using chronic kidney disease epidemiology collaboration equation; GGT, gamma‐glutamyl transferase; HbA1c, hemoglobin A1C; HDL‐C, high‐density lipoprotein cholesterol; LDL‐C, low‐density lipoprotein cholesterol; SD, standard deviation; T2DM, Type 2 diabetes mellitus; TGs, triglycerides.

^a^ Mean (SD) or *n* (%).

### 3.2 MASLD prevalence and associated factors

**Fig. 2:**
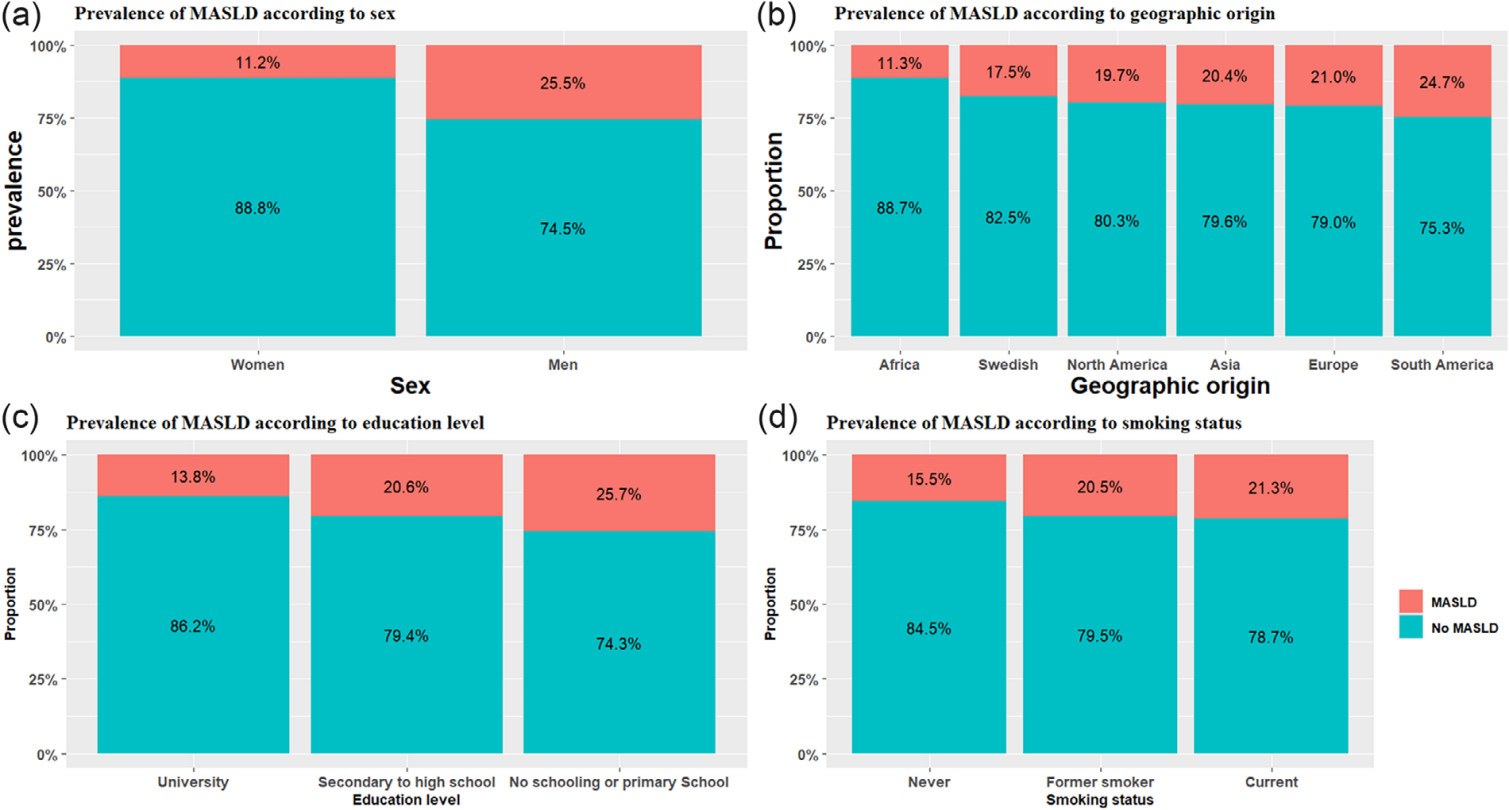
Metabolic dysfunction–associated steatotic liver disease (MASLD) prevalence stratified by (a) sex, (b) geographic origin, (c) education level, and (d) smoking status.

**Fig. 3:**
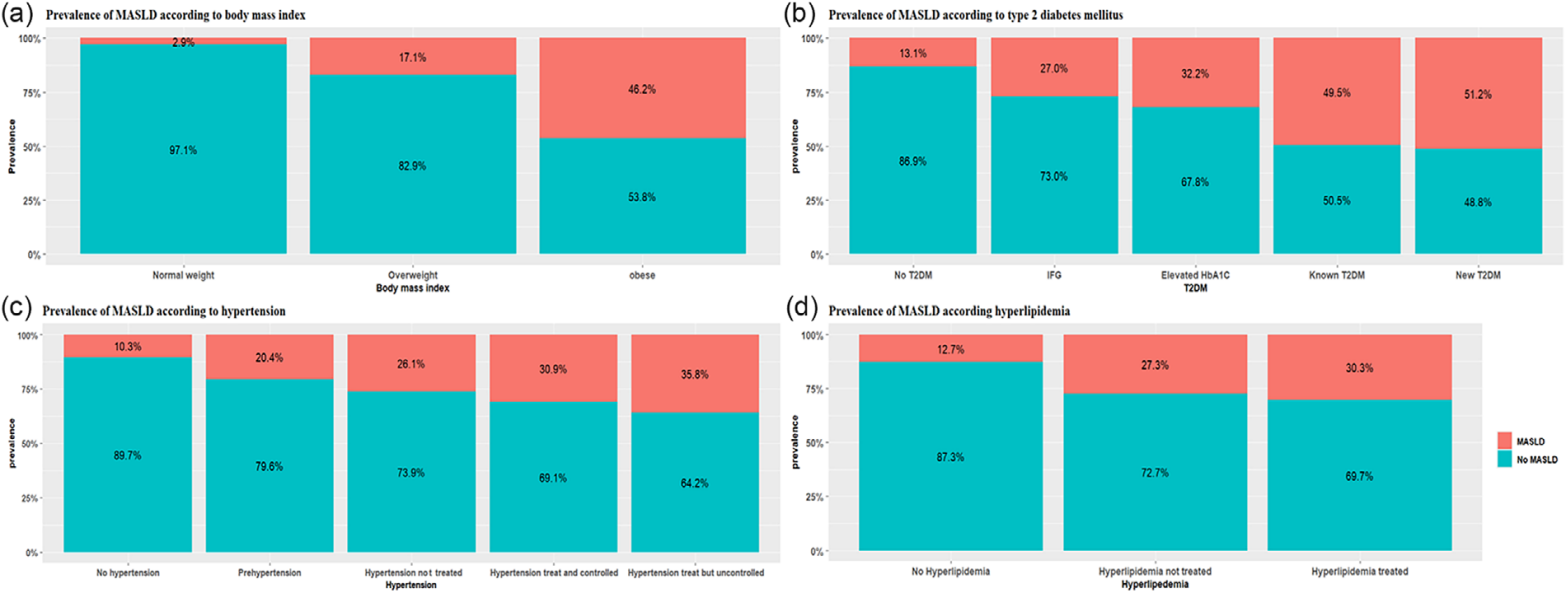
Metabolic dysfunction–associated steatotic liver disease (MASLD) prevalence stratified by (a) body mass index, (b) Type 2 diabetes mellitus, (c) hypertension, and (d) hyperlipidemia.

**Fig. 4:**
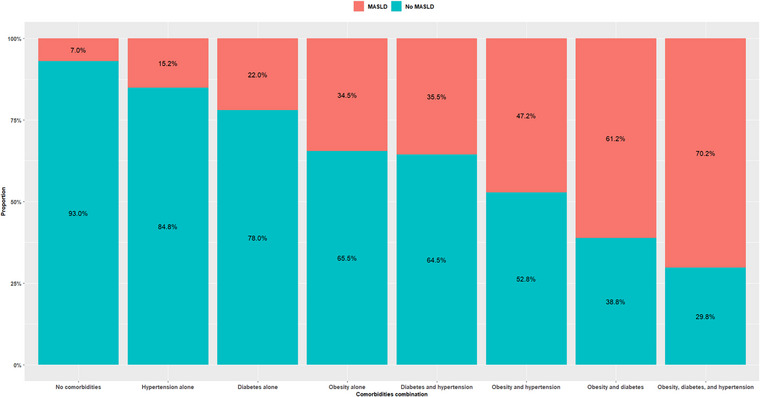
Metabolic dysfunction–associated steatotic liver disease (MASLD) prevalence according to the cardiometabolic comorbidities (obesity, Type 2 diabetes mellitus, and hypertension) combination.

Among the 27,236 SCAPIS participants with complete data, 4933 individuals (18.1%) met the criteria for MASLD, defined by a liver attenuation of less than 48 HU on non‐contrast CT imaging combined with the presence of at least one cardiometabolic risk factor. MASLD prevalence was significantly higher in men compared to women (25.5% vs. 11.2%; *p *< 0.001). A geographic variation was observed: MASLD prevalence was highest among individuals of South American origin (24.7%), followed by those of European (21.0%), Asian (20.4%), North American (19.7%), and Swedish origin (17.5%), and was lowest among individuals of African origin (11.3%) (Fig. 2). MASLD prevalence was inversely proportionate with the education level and was associated with smoking status (15.5% vs. 20.5% vs. 21.3%, respectively, in those who never smoked, former smokers, and the current smoker subgroups) (Fig. 2). MASLD prevalence was 2.9% among normal‐weight individuals, 17.1% among those classified as overweight, and 46.2% in individuals with obesity. MASLD was also more prevalent among participants with T2DM (45.6%), hypertension (29.3%), and hyperlipidemia (28.0%) (Fig. 3 and Table S2), and the prevalence increased with higher BMI, more severe in T2DM and hypertension (Fig. 3). The burden of MASLD increased with the number of coexisting metabolic conditions: The prevalence was 7.0% in participants without either obesity, diabetes, or hypertension and peaked at 70.2% in individuals with all three conditions (Fig. 4). When dyslipidemia was additionally excluded, the prevalence of MASLD among participants without obesity, diabetes, or hypertension decreased from 7.0% to 4.4% (Fig. S1). In univariable (Table S3) and multivariable logistic regression models, several factors were significantly associated with higher odds of MASLD (Table 2 and Table S3). These included male sex (aOR: 2.16; 95% CI: 1.97, 2.36), older age (aOR per year: 1.04; 95% CI: 1.03, 1.05), obesity alone (aOR: 5.56; 95% CI: 4.89, 6.31), T2DM alone (aOR: 2.66; 95% CI: 2.13, 3.33), and hypertension alone (aOR: 1.78; 95% CI: 1.59, 1.99) (Table 2, Model 3). The combined presence of obesity, diabetes, and hypertension was associated with a 12‐fold increased risk of MASLD (aOR: 17.1; 95% CI: 14.0, 20.9) compared to participants with none of these conditions. Lifestyle factors also played a significant role. Physical activity was associated with a dose‐dependent protective effect: Compared with sedentary individuals, those who exercised irregularly or moderately had lower odds of MASLD (aOR: 0.80; 95% CI: 0.71, 0.89), and the protective association strengthened with regular and more intense activity (aOR: 0.49; 95% CI: 0.43, 0.56 for moderate regular exercise; aOR: 0.34; 95% CI: 0.29, 0.41 for regular vigorous exercise). Conversely, certain lifestyle habits were associated with increased MASLD risk, including soda consumption of two or more times per day compared to no consumption (aOR: 1.49; 95% CI: 1.19, 1.88), former smoking (aOR: 1.21; 95% CI: 1.11, 1.32), and current smoking (aOR: 1.31; 95% CI: 1.15, 1.49) compared to never smoking. Notably, high coffee consumption, defined as four or more cups per day, was associated with increased odds of MASLD (aOR: 1.73; 95% CI: 1.41, 2.13), as shown in Table 2 (Model 3).

**Table 2:** Multivariable logistic regression analyses of metabolic dysfunction–associated steatotic liver disease (MASLD) risk factors (reference group: No MASLD)

Characteristics	OR	95% CI	*p*‐value
**Model 2**			
Age, year	1.04	1.03, 1.05	<0.001
Sex			
Women	1.0	Reference	
Men	2.17	1.98, 2.37	<0.001
Geographic origin			
Sweden	1.0	Reference	
Europe	0.80	0.70, 0.92	0.001
North America	1.02	0.45, 2.17	0.962
South America	1.09	0.73, 1.60	0.676
Asia	0.72	0.57, 0.89	0.003
Africa	0.48	0.24, 0.88	0.023
Education level			
Primary education	1.0	Reference	
Secondary education	1.03	0.90, 1.18	0.642
Tertiary education	0.97	0.84, 1.12	0.679
BMI class			
Normal weight	1.0	Reference	
Overweight	3.82	3.31, 4.44	<0.001
Obesity	12.9	11.1, 15.1	<0.001
T2DM			
No T2DM	1.0	Reference	
IFG	1.57	1.41, 1.75	<0.001
New T2DM	3.26	2.67, 3.97	<0.001
Treated T2DM	3.21	2.67, 3.86	<0.001
Elevated HbA1c	1.91	1.53, 2.37	<0.001
Hypertension class			
No HBP	1.0	Reference	
Prehypertension unknown	1.45	1.28, 1.65	<0.001
HBP not treated	1.66	1.48, 1.86	<0.001
HBP treated and controlled	1.69	1.50, 1.90	<0.001
HBP treated but uncontrolled	1.89	1.55, 2.31	<0.001
Hyperlipidemia			
No hyperlipidemia	1.0	Reference	
Hyperlipidemia treated	0.83	0.72, 0.97	0.017
Hyperlipidemia not treated	1.67	1.53, 1.82	<0.001
ALT, U/L	1.05	1.05, 1.06	<0.001
AST, U/L	0.98	0.97, 0.99	<0.001
Exercise			
Sedentary	1.0	Reference	
Moderate but not regular	0.80	0.71, 0.90	<0.001
Moderate but regular	0.52	0.45, 0.59	<0.001
Regular exercise	0.38	0.31, 0.46	<0.001
Coffee consumption			
No or less than 1 time a week	1.0	Reference	
Once a day	1.70	1.18, 2.43	0.004
2–3 times a day	1.32	1.15, 1.51	<0.001
4 times a day or more often	1.68	1.35, 2.08	<0.001
Soda intake			
No or less than times per week	1.0	Reference	
Once a day	1.12	1.00, 1.26	0.057
2 times a day or more often	1.44	1.14, 1.81	0.002
Smoking status			
Never	1.0	Reference	
Former smoker	1.24	1.13, 1.36	<0.001
Current	1.32	1.15, 1.50	<0.001
Alcohol consumption, g/d	1.02	1.02, 1.03	<0.001
**Model 3**			
Age	1.04	1.03, 1.05	<0.001
Sex			
Women	1.0	Reference	
Men	2.16	1.97, 2.36	<0.001
Geographic origin			
Swedish	1.0	Reference	
Europe	0.88	0.77, 1.01	0.064
North America	1.08	0.47, 2.30	0.848
South America	1.24	0.84, 1.82	0.277
Asia	0.86	0.70, 1.07	0.175
Africa	0.55	0.29, 1.04	0.065
Education level			
Primary education	1.0	Reference	
Secondary education	1.00	0.88, 1.15	0.976
Tertiary education	0.89	0.78, 1.03	0.119
Hyperlipidemia	1.51	1.39, 1.63	<0.001
Comorbidities			
No comorbidities	1.0	Reference	
T2DM alone	2.66	2.13, 3.33	<0.001
HBP alone	1.78	1.59, 1.99	<0.001
Obesity alone	5.56	4.89, 6.31	<0.001
T2DM and HBP	4.49	3.70, 5.47	<0.001
Obesity and T2DM	15.8	12.1, 20.7	<0.001
Obesity and HBP	7.59	6.70, 8.58	<0.001
Obesity, T2DM, and HBP	17.1	14.0, 20.9	<0.001
ALT	1.06	1.05, 1.06	<0.001
AST	0.98	0.97, 0.98	<0.001
Exercise			
Sedentary	1.0	Reference	
Moderate but not regular	0.80	0.71, 0.89	<0.001
Moderate but regular	0.49	0.43, 0.56	<0.001
Regular exercise	0.34	0.43, 0.56	<0.001
Coffee consumption			
No or less 1 time a week	1.0	Reference	
Once a day	1.77	1.25, 2.52	0.001
2–3 times a day	1.28	1.12, 1.46	<0.001
4 times a day or more often	1.73	1.41, 2.13	<0.001
Soda intake			
No or less than times per week	1.0	Reference	
Once a day	1.04	0.93, 1.17	0.472
2 times a day or more often	1.49	1.19, 1.88	0.001
Smoking status			
Never	1.0	Reference	
Former smoker	1.21	1.11, 1.32	<0.001
Current	1.22	1.07, 1.39	0.003
Alcohol consumption, g/d	1.02	1.02, 1.03	<0.001

*Note*: Unadjusted estimates and estimates from Model 1 are presented in Table S3. Model 2: BMI in three classes (normal weight; overweight; obese); T2DM status in six modalities (no T2DM vs. IFG vs. new T2DM vs. T2DM under treatment and controlled vs. elevated HbA1c); hypertension status in five modalities (no hypertension vs. prehypertension vs. hypertension not treated vs. hypertension treated and controlled vs. hypertension treated but uncontrolled); hyperlipidemia in three classes (no hyperlipidemia vs. hyperlipidemia treated vs. hyperlipidemia untreated); and further adjusted for age, sex, geographic origin, education level, AST, ALT, gamma‐glutamyl transferase, sport practice, coffee consumption, soda intake, smoking, and residual alcohol consumption (g/day). Model 3: comorbidities (obesity: yes/no, T2DM: yes/no, and hypertension: yes/no) combination, and further adjusted for age, sex, geographic origin, education level, hyperlipemia (yes/no), AST, ALT, gamma‐glutamyl transferase, sport practice, coffee consumption, soda intake, smoking, and residual alcohol consumption. All multivariable models that included both BMI and waist circumference were not retained based on the AIC criterion. Among the models evaluated, those including BMI without waist circumference had lower AIC values than models, including waist circumference without BMI.

Abbreviations: ALT, alanine transaminase; AST, aspartate aminotransferase; BMI, body mass index; CI, confidence intervals; g/d, gram/day; HbA1c, hemoglobin A1C; HBP, high blood pressure (hypertension); IFG, impaired fasting glucose; OR, odds ratios; T2DM, type 2 diabetes mellitus.

### 3.3 Estimating liver fibrosis prevalence among individuals with MASLD and associated risk factors

Among the 4933 participants with MASLD, 1225 (24.8%) were classified as at risk for advanced liver fibrosis, compared to 1399 (6.3%) of the 22,303 participants without MASLD (*p* < 0.001). The general characteristics of the individuals with MASLD with and without risk for advanced liver fibrosis according to this dAAR cutoff are shown in Table 3. A high dAAR score was observed in men with MASLD (72%) and in individuals with obesity (56%), hypertension (63%), or T2DM (28%). The prevalence of estimated advanced fibrosis risk increased modestly with adiposity: 20.7% among normal‐weight MASLD participants, 24.5% among those overweight, and 25.6% among individuals with obesity. Similarly, the estimated prevalence of advanced fibrosis was higher among participants with metabolic comorbidities: It affected 29% of those with diabetes, 28.3% with hypertension, and 27.7% with hyperlipidemia (Table S2). Associations between clinical and biochemical variables and the presence of advanced liver fibrosis were assessed using both univariable (Table S4) and multivariable (Table 4) logistic regression analyses. The multivariable Model 1 was adjusted for sex, geographic origin, education level, obesity, T2DM, hypertension, hyperlipidemia, and lifestyle factors (physical activity, alcohol intake, smoking, and coffee and soda consumption).

**Table 3:** General characteristics of metabolic dysfunction–associated steatotic liver disease (MASLD) patients according to advanced liver fibrosis estimated with dynamic aspartate‐to‐alanine aminotransferase ratio (dAAR)

Characteristics^a^	All MASLD participants *N* = 4933	MASLD with dAAR ≤1.5708 (no advanced fibrosis) *N* = 3565	MASLD with dAAR >1.5708 (advanced fibrosis) *N* = 1225
Sex			
Women	1569 (31.8%)	1163 (32.6%)	336 (27.4%)
Men	3364 (68.2%)	2402 (67.4%)	889 (72.6%)
Geographic origin			
Swedish	3925 (79.6%)	2807 (78.7%)	1003 (81.9%)
Europe	704 (14.3%)	528 (14.8%)	154 (12.6%)
North America	13 (0.3%)	7 (0.2%)	5 (0.4%)
South America	63 (1.3%)	45 (1.3%)	16 (1.3%)
Asia	211 (4.3%)	164 (4.6%)	44 (3.6%)
Africa	17 (0.3%)	14 (0.4%)	3 (0.2%)
Education level			
Primary education	606 (12.7%)	444 (12.9%)	145 (12.2%)
Secondary education	2488 (52.1%)	1796 (52.1%)	617 (52.0%)
Tertiary education	1678 (35.2%)	1209 (35.1%)	424 (35.8%)
Height, cm	174.7 (9.7)	174.7 (9,7)	174.9 (9.4)
Weight, kg	94.7 (16.3)	94.4 (16.3)	95.4 (15.8)
Waist circumference, cm	107.2 (11.3)	106.8 (11.4)	108.3 (10.8)
Hip circumference, cm	108.2 (9.9)	108.2 (10.1)	107.8 (8.9)
BMI, kg/m^2^	31.0 (4.7)	30.9 (4.8)	31.1 (4.4)
BMI class			
Normal weight	275 (5.6%)	214 (6.0%)	57 (4.7%)
Overweight	1977 (40.2%)	1437 (41.0%)	485 (39.7%)
Obesity	2661 (54.2%)	1896 (53.0%)	681 (55.6%)
Systolic blood pressure, mmHg	133.0 (16.0)	132.4 (16.0)	134.8 (15.7)
Diastolic blood pressure, mmHg	81.9 (9.9)	81.5 (9.9)	83.0 (9.5)
Hypertension, yes	2742 (55.6%)	1892 (53.1%)	776 (63.3%)
Hypertension treatment, yes	1665 (33.8%)	1116 (31.3%)	494 (40.3%)
T2DM, yes	1209 (25.0%)	826 (23.2%)	338 (27.6%)
		436 (12.2%)	157 (12.8%)
Known T2DM and treated with drugs	474 (76.5%)	331 (75.9%)	124 (79.9%)
Glucose, mmol/L	6.46 (1.7)	6.4 (1.7)	6.6 (1.8)
HbA1c, %	5.8 (0.9)	5.8 (0.9)	5.8 (0.9)
Insulin, mIU/L	13 (39.2)	12.5 (41.5)	14.5 (31.5)
GGT, IU/L	50.8 (69.0)	41.0 (30.2)	79.0 (121.9)
Total cholesterol, mmol/L	5.4 (1.1)	5.4 (1.1)	5.4 (1.2)
LDL‐C, mmol/L	3.5 (1.0)	3.5 (1.0)	3.4 (1.0)
LDL‐C, mmol/L	1.3 (0.4)	1.3 (0.4)	1.3 (0.4)
TGs, mmol/L	1.8 (1.3)	1.7 (1.0)	1.9 (1.6)
Hyperlipidemia, yes	2681 (54.5%)	1884 (52.9%)	722 (59.1%)
Creatinine, mmol/L	79.9 (16.1)	79.8 (16.2)	80.6 (15.5)
eGFR CKD EPI mL/min/m^2^	90.0 (12.3)	90.2 (12.4)	89.4 (12.0)
Smoking status			
Never	2121 (45.5%)	1532 (45.5%)	531 (44.8%)
Former smoker	1966 (41.3%)	1415 (41.1%)	500 (42.2%)
Current	676 (14.2%)	493 (14.3%)	155 (13.1%)

Abbreviations: ALT, alanine transaminase; AST, aspartate aminotransferase; BMI, body mass index; eGFR CKD EPI, estimated glomerular filtration rate using chronic kidney disease epidemiology collaboration equation; GGT, gamma‐glutamyl transferase; HbA1c, hemoglobin A1C; HDL‐C, high‐density lipoprotein cholesterol; LDL‐C, low‐density lipoprotein cholesterol; SD, standard deviation; T2DM, type 2 diabetes mellitus; TGs, triglycerides.

^a^ *n* (%); Mean (SD)

**Table 4:** Multivariable analyses of liver fibrosis (estimated by dynamic aspartate‐to‐alanine aminotransferase ratio [dAAR]) determinants in participants with metabolic dysfunction–associated steatotic liver disease (MASLD) according to comorbidity combination

Liver fibrosis risk in participants with MASLD (dAAR >1.5708)			
	OR	95% CI	*p*‐value
**Multivariable analyses Model 1**			
Sex, men	1.20	1.02, 1.42	0.031
Geographic origin			
Swedish	1.0	Reference	
Europe	0.86	0.68, 1.08	0.199
North America	2.25	0.70, 7.22	0.174
South America	1.27	0.69, 2.34	0.449
Asia	0.72	0.48, 1.08	0.108
Africa	0.82	0.23, 2.97	0.767
Education level			
Primary education	1.0	Reference	
Secondary education	1.06	0.84, 1.32	0.633
Tertiary education	1.09	0.86, 1.38	0.463
Obesity, yes	1.07	0.92, 1.23	0.378
T2DM, yes	1.24	1.06, 1.46	0.009
Hypertension, yes	1.44	1.24, 1.66	<0.001
Hyperlipidemia, yes	1.22	1.06, 1.40	0.007
Exercise			
Sedentary	1.0	Reference	
Moderate but not regular	1.07	0.90, 1.28	0.447
Moderate but regular	1.01	0.81, 1.26	0.937
Regular exercise	0.92	0.64, 1.31	0.638
Coffee consumption			
No or less than 1 time a week	1.0	Reference	
Once a day	1.21	0.72, 2.04	0.476
2–3 times a day	1.24	1.02, 1.54	0.045
4 times a day or more often	1.35	0.97, 1.87	0.072
Soda intake			
No or less than times per week	1.0	Reference	
Once a day	1.00	0.82, 1.22	0.995
2 times a day or more often	0.90	0.60, 1.33	0.581
Smoking status			
Never	1.0	Reference	
Former smoker	0.96	0.83, 1.12	0.609
Current smoker	0.92	0.74, 1.15	0.458
Alcohol consumption, g/d	1.02	1.01, 1.03	<0.001
**Multivariable analyses Model 2**			
Sex, men	1.15	0.97, 1.38	0.110
Geographic origin			
Swedish	1.0	Reference	
Europe	0.86	0.68, 1.09	0.206
North America	2.29	0.71, 7.38	0.165
South America	1.29	0.70, 2.38	0.420
Asia	0.75	0.50, 1.13	0.164
Africa	0.84	0.23, 3.05	0.795
Education level			
Primary education	1.0	Reference	
Secondary education	1.06	0.84, 1.32	0.628
Tertiary education	1.10	0.87, 1.39	0.446
Waist circumference	1.01	1.00, 1.02	0.105
Hyperlipidemia, yes	1.21	1.05, 1.40	0.007
Comorbidities’ combination			
No comorbidities	1.0	Reference	
T2DM alone	1.24	0.80, 1.93	0.330
HBP alone	1.46	1.16, 1.83	0.001
Obesity alone	0.88	0.67, 1.16	0.378
T2DM and HBP	1.59	1.14, 2.21	0.006
Obesity and T2DM	1.51	1.02, 2.23	0.037
Obesity and HBP	1.41	1.10, 1.82	0.007
Obesity, T2DM, and HBP	1.61	1.19, 2.17	0.002
Exercise			
Sedentary	1.0	Reference	
Moderate but not regular	1.05	0.87, 1.27	0.594
Moderate but regular	0.92	0.64, 1.31	0.632
Regular exercise	0.96	0.77, 1.21	0.735
Coffee consumption			
No or less than 1 time a week	1.0	Reference	
Once a day	1.20	0.69, 2.01	0.505
2–3 times a day	1.28	1.03, 1.59	0.024
4 times a day or more often	1.37	0.97, 1.90	0.065
Soda intake			
No or less than times per week	1.0	Reference	
Once a day	1.01	0.83, 1.23	0.903
2 times a day or more often	0.90	0.60, 1.32	0.607
Smoking status			
Never	1.0	Reference	
Former smoker	0.96	0.82, 1.12	0.587
Current smoker	0.92	0.73, 1.15	0.456
Alcohol consumption, g/d	1.02	1.01, 1.03	<0.001

*Note*: Restricted multivariable model was adjusted for sex, geographic origin, education level, obesity (yes/no), T2DM (yes/no), hypertension (yes/no), and hyperlipidemia (yes/no). Full multivariable model was adjusted for comorbidities (obesity: yes/no, T2DM: yes/no, and hypertension: yes/no) combination, and further adjusted for sex, geographic origin, education level, hyperlipidemia (yes/no), waist circumference, hyperlipidemia, sport practice, coffee consumption, soda intake, smoking, and residual alcohol consumption.

Abbreviations: BMI, body mass index; CI, confidence intervals; g/d, gram/day; HBP, high blood pressure (hypertension); OR, odds ratios; T2DM, type 2 diabetes mellitus.

Model 2 included all covariates from Model 1, with additional adjustment for waist circumference, and individual comorbidities (obesity, T2DM, and hypertension) were replaced by a mutually exclusive composite variable representing their combinations (Table 4).

Several cardiometabolic factors were independently associated with greater odds of advanced fibrosis risk. Male sex was associated with a 20% increase in risk (aOR: 1.20; 95% CI: 1.02, 1.42), whereas hypertension (aOR: 1.44; 95% CI: 1.24, 1.66), T2DM (aOR: 1.24; 95% CI: 1.06, 1.46), hyperlipidemia (aOR: 1.22; 95% CI: 1.06, 1.40), and alcohol consumption (aOR per gram/day: 1.02; 95% CI: 1.01, 1.03) also conferred an elevated risk (Table 4, Model 1). Further stratified analyses confirmed the consistency of these associations, particularly for male sex and hypertension, across MASLD subgroups defined by different combinations of metabolic comorbidities (Table 4).

## 4 Discussion

This large population‐based study provides robust epidemiological evidence on the burden of MASLD in Sweden. Using CT imaging with a liver attenuation threshold of <48 HU, the prevalence of MASLD was estimated at 18%. Although this figure appears lower than global pooled estimates ranging from 30% to 38% [1], it must be interpreted in light of age distribution and methodology. In a study conducted in Uppsala, Sweden, which included 308 participants and used magnetic resonance imaging (MRI) for liver fat quantification, the prevalence of NAFLD was estimated at 22.7%, slightly higher than the prevalence observed in our study [11]. The SCAPIS cohort includes participants aged 50–64 years, a range in which MASLD prevalence tends to be higher than in younger populations. Indeed, comparative European cohorts, such as CONSTANCES and the UK Biobank cohorts, have reported higher age‐adjusted prevalence in this age group [18, 19, 20]. These discrepancies are also likely influenced by differences in diagnostic modality with CT being less sensitive to mild hepatic steatosis compared to MRI‐based techniques or composite biochemical indices [21, 22, 23]. In the current study, we also use a rather high HU cutoff to define hepatic steatosis, which may contribute to an underestimation of its prevalence. Additionally, a recent systematic review and meta‐analysis by Haghshomar et al. reported high specificity but only moderate sensitivity for detecting ≥5% hepatic fat, with improved, though still imperfect, performance for moderate steatosis (≥20%–33% fat) [24]. Consequently, some individuals with true steatosis may be misclassified as non‐steatotic, leading to contamination of the “non‐MASLD” reference group and underestimation of MASLD prevalence. Alternative imaging‐based definitions, such as the liver‐to‐spleen attenuation ratio or direct liver–spleen structure comparisons, have also been used in previous research [25]; however, such measures were not available in the SCAPIS dataset. Additionally, the volunteer nature of SCAPIS may introduce healthy participant bias, even if we cannot exclude the alternative hypothesis that the findings of previous studies may have been influenced by selection bias.

A pronounced sex disparity was observed: MASLD prevalence was more than twice as high in men (25.5%) than in women (11.2%), a pattern consistently reported in prior large‐scale studies [18, 19, 20]. This male predominance is likely multifactorial, reflecting higher visceral adiposity, different sex hormone profiles, particularly the hepatoprotective effects of estrogens, and differences in diet and alcohol use patterns [26]. Age and cardiometabolic risk factors were independently and strongly associated with MASLD. Obesity showed the highest adjusted odds (aOR: 5.56), followed by T2DM (aOR: 2.66) and hypertension (aOR: 1.78). The cumulative effect of multiple comorbidities was striking, with individuals harboring all three conditions experiencing more than 12‐fold increased odds of MASLD. These results are in strong concordance with the existing literature highlighting insulin resistance, lipotoxicity, and chronic low‐grade inflammation as key drivers of MASLD pathophysiology [27, 28, 29].

Notably, approximately 4.4% of participants exhibited MASLD despite the absence of obesity, diabetes, hyperlipidemia, or hypertension, indicating that a small but non‐negligible proportion of hepatic steatosis occurs outside the classical metabolic risk profile. This finding likely reflects etiologic heterogeneity and may involve unmeasured or incompletely captured factors, such as genetic susceptibility, alcohol exposure below reporting thresholds, ethnic‐specific risk patterns, or other environmental and biological determinants, warranting further investigation.

Modifiable behavioral risk factors were also significantly associated with MASLD. Vigorous physical activity conferred a robust protective effect (aOR: 0.34), and even moderate or irregular activity showed a measurable reduction in disease odds. These findings echo those from previous studies demonstrating the beneficial impact of physical activity, both aerobic and resistance training, on hepatic fat content, liver enzyme levels, and histological outcomes in MASLD [30, 31, 32]. Importantly, recent interventional studies have suggested that even modest improvements in physical activity, without significant weight loss, can yield clinically meaningful reductions in intrahepatic fat, thus positioning exercise as a cornerstone of MASLD management [33]. This further strengthens the evidence for exercise as the first‐line treatment in MASLD [34]. Conversely, higher consumption of sugar‐sweetened beverages (aOR: 1.49), current or former smoking (aORs: 1.21–1.22), and high coffee intake (≥4 cups/day; aOR: 1.73) were associated with increased odds of MASLD. Although sugar intake and smoking are well‐established MASLD risk factors [35, 36, 37, 38], the association with coffee is more surprising, given previous reports of hepatoprotective effects [39, 40, 41]. This discordance may reflect residual confounding, differences in coffee preparation (e.g., filtered vs. unfiltered), or reverse causation. Further longitudinal and mechanistic studies are needed to clarify this relationship.

Ethnic and geographic disparities were evident, with a higher MASLD prevalence among individuals of South American (24.7%) and European (21.0%) ancestry and a lower prevalence in those of African descent (11.3%). These patterns align with data wide evidence in current literature [1, 42, 43, 44, 45] and may reflect differences in genetic predisposition (e.g., *PNPLA3* and *TM6SF2* variants) [46, 47], dietary practices [47, 48, 49, 50], and physical activity behaviors [51, 52]. The lower prevalence in individuals of African ancestry has been previously attributed to a lower propensity for hepatic fat accumulation despite comparable levels of visceral adiposity, a phenomenon that may be partially genetically mediated [53, 54]. Taken together, our findings underscore a substantial, yet underestimated, burden of MASLD in Sweden.

In our study, 24.8% were classified as at risk for advanced liver fibrosis. The estimated prevalence of advanced fibrosis was 29% among participants with T2DM, 28.3% among those with hypertension, and 27.7% among those with hyperlipidemia. The fibrosis prevalence observed in our study, including in individuals with T2DM, hypertension, and hyperlipidemia, exceeds that reported in several recent epidemiological and community‐based studies [18, 55, 56, 57], which have generally relied on alternative noninvasive tools or imaging‐based fibrosis assessment, and therefore warrant careful interpretation. Although most methods to investigate liver fibrosis prevalence have modest performance, these data suggest a considerably higher risk for fibrosis in an MASLD population compared to individuals without MASLD. Integrating liver health assessment into routine cardiometabolic evaluations in high‐risk groups may improve early detection and intervention, although more effective methods such as vibration‐controlled transient elastography (VCTE) might be better suited. Our data suggest this to be more important in individuals with several components of the metabolic syndrome. This is further supported by previous data from our group showing that in patients with T2DM, additional traits of the metabolic syndrome confer a higher risk of major adverse liver outcomes [58].

The high prevalence of estimated advanced liver fibrosis risk observed in our study compared to prior population‐based studies may be partly explained by the age distribution of our cohort, which included individuals aged 50–64 years, a group at a greater risk for fibrosis progression. Additionally, we focused on participants with more severe hepatic steatosis (estimated ≥30% liver fat content), whereas earlier studies often included individuals with milder forms of MASLD, typically defined by a liver fat threshold of 5%. This selection of a higher risk population may have contributed to the high fibrosis estimates in our analysis. Fibrosis assessment was restricted to the dAAR score due to the absence of biopsy, imaging, and key laboratory data, such as platelet counts in SCAPIS. Although dAAR offers only moderate diagnostic accuracy relative to established scores like FIB‐4 and imaging modalities, particularly near fibrosis thresholds [17], it remains the sole feasible metric in this context. This limitation may introduce a misclassification, potentially inflating or underestimating fibrosis prevalence; therefore, our estimates should be interpreted as approximate.

Male sex and cardiometabolic comorbidities, including obesity, T2DM, hyperlipidemia, and notably hypertension, were independently associated with increased odds of a high dAAR score. Among these, hypertension emerged as the strongest predictor (aOR: 1.44; 95% CI: 1.24–1.66), a finding that corroborates and extends evidence from other population‐based studies [18, 59, 60, 61, 62, 63, 64]. Although previous research has consistently demonstrated associations between metabolic traits and liver fibrosis, our study uniquely quantifies the additive and independent contribution of hypertension even in the absence of obesity or diabetes, thus identifying a high‐risk subgroup that is likely overlooked under current clinical guidelines. Even though self‐reported data on alcohol consumption were available, no objective biomarker such as phosphatidylethanol was available. Hence, alcohol consumption, which can also lead to hypertension, is likely a residual confounder in this analysis.

Current recommendations from both AASLD [65] and EASL [34] advise noninvasive liver fibrosis screening in patients with T2DM, obesity with at least one additional cardiometabolic risk factor, or repeatedly elevated aminotransferases. Our data together with recent studies in T2DM [18, 55, 56, 57, 58] suggest that hypertension [18, 59, 60, 61, 62, 63, 64] may be included as a key risk factor for liver fibrosis screening. Importantly, even modest alcohol consumption was associated with a disproportionate increase in both MASLD and fibrosis risk among individuals with MASLD, reinforcing the concept that there may be no safe alcohol threshold in this population and underscoring the importance of alcohol abstinence in minimizing both MASLD and fibrogenesis and informing clinical practice and public health policy. These data may aid clinical and public health strategies for MASLD in Sweden and similar high‐income countries.

This study's major strength lies in its use of a large, well‐characterized, and phenotypically rich cohort, enabling robust estimation of the prevalence of MASLD in a middle‐aged Swedish cohort. Additionally, the comprehensive assessment of cardiometabolic comorbidities allowed for refined multivariable modeling to disentangle the independent contributions of specific risk factors. Nonetheless, several limitations must be acknowledged. First, hepatic steatosis was assessed using CT imaging with a liver attenuation threshold of <48 HU, a cutoff commonly used to identify clinically relevant, predominantly moderate‐to‐severe steatosis. Because of the limited sensitivity of CT [24], particularly for mild steatosis and incompletely for moderate disease, some individuals with true hepatic steatosis may have been misclassified as non‐steatotic. This likely resulted in contamination of the “non‐MASLD” reference group and attenuation of between‐group contrasts. Such non‐differential misclassification would be expected to bias associations toward the null rather than generate spurious findings, indicating that observed associations are likely conservative.

In addition, steatosis may regress with advancing fibrosis, particularly during the transition from advanced fibrosis (F3) to cirrhosis (F4), a phenomenon often referred to as “burnt‐out” steatosis [66, 67]. Consequently, individuals with the most advanced fibrotic phenotypes may exhibit low hepatic fat content on imaging and be misclassified as non‐steatotic despite biologically advanced disease, further contributing to underestimation of disease burden at the severe end of the spectrum. The use of a higher attenuation threshold therefore reflects a deliberate trade‐off favoring specificity over sensitivity, enriching the identified MASLD group for metabolically active, steatosis‐rich disease. Accordingly, our findings primarily pertain to moderate‐to‐severe MASLD and should be interpreted as conservative estimates of prevalence and associations within the context of these methodological and biological constraints. Second, the cross‐sectional design inherently limits causal inference and precludes the assessment of temporal relationships between risk factors and fibrosis progression. Third, the original SCAPIS data collection was not focused on liver‐related aspects, which is why MASLD was defined using imaging‐derived liver fat content in combination with metabolic criteria and no direct fibrosis quantification methods, such as VCTE, were included. Fourth, the restriction of our analysis to participants with moderate to severe steatosis using the 48 Hounsfield cutoff may have led to an underestimation of the true prevalence of MASLD in this population. However, using a higher cutoff may instead have overestimated the prevalence. Moreover, although the dAAR score is validated as an estimation of liver fibrosis, it remains a surrogate marker of fibrosis and may be susceptible to misclassification. Residual confounding remains a potential concern despite adjustment for multiple covariates. Unmeasured factors, including genetic and epigenetic variations, gut microbiota composition, and the use of hepatotoxic or potentially antifibrotic medications, may have influenced both fibrosis risk and its assessment. Finally, the cohort's age range (50–64 years) may limit generalizability to other ages and may partly explain the high estimated prevalence of advanced fibrosis observed relative to previous studies with broader age distributions.

## 5 Conclusion

This large population‐based study provides one of the first national estimates of MASLD prevalence and fibrosis risk in Sweden using standardized CT imaging and updated metabolic criteria. Nearly one in five middle‐aged adults is affected by MASLD, with the burden most prevalent in those with obesity, T2DM, hypertension, and male sex. Importantly, nearly one in four people with MASLD was at risk of advanced fibrosis, indicating an under‐recognized public health challenge. Future studies to investigate adverse events are underway. Our findings emphasize the need for studies to improve prevention and better determine where and how case finding for fibrosis due to MASLD should be conducted.

## Author contributions

*Study conception and design*: Oumarou Nabi, Hannes Hagström. *Acquisition of data*: Hannes Hagström. *Statistical analysis*: Oumarou Nabi. *Analysis and interpretation of data*: Oumarou Nabi and Hannes Hagström. *Drafting of manuscript*: Oumarou Nabi and Hannes Hagström. *Critical revision*: All the coauthors. *Guarantor of article*: Hannes Hagström. All authors approved the final version of the article, including the authorship list.

## Conflict of interest statement

**Joel Kullberg** is the cofounder, shareholder, and part‐time employee of Antaros Medical. **Tomas Jernberg** institutional grant from MSD is not related to this manuscript. **Daniel P. Andersson** has received nonfinancial support from COVIS Pharma (study drug donation) as a principal investigator in another study. **Daniel P. Andersson** is site principal investigator for Ionis 678354 for Ionis Pharmaceuticals, Inc. **Daniel P. Andersson** has part‐time employment at Werlabs AB. None of these studies or **Daniel P. Andersson**’s employment is related to the present work. **Hannes Hagström's** institutions have received research funding from Astra Zeneca, Echosens, Gilead, Intercept, MSD, Novo Nordisk, Takeda, and Pfizer. He has served as a consultant, speaker, or on advisory boards for Astra Zeneca, Boehringer Ingelheim, Bristol Myers‐Squibb, GSK, Echosens, Ipsen, MSD, and Novo Nordisk and has been part of hepatic events adjudication committees for Arrowhead, Boehringer Ingelheim, KOWA, and GW Pharmaceuticals. Other authors declare no competing interests related to this manuscript.

The manuscript has been handled by an external editor, Anders Waldenström, Emeritus professor, Department of Public Health and Clinical Medicine, Umeå University, SE 90187 Umeå, Sweden.

## Funding information

The study was supported by grants from The Swedish Research Council, Region Stockholm (CIMED and clinical researcher award to Hannes Hagström) and The Swedish Heart and Lung Foundation. **Daniel P. Andersson** was supported by the Stockholm County Council, CIMED at Karolinska Institutet, and the Swedish Society of Medicine. The funders had no role in the study design, conduct, collection, management, analysis, interpretation of data, writing or reviewing the manuscript, or decision to submit the manuscript for publication.

## Ethical considerations

The SCAPIS study was approved by the Swedish Ethical Review Authority (Reference Number: diary number 2010‐228‐31M, with addendum 2011‐02‐21, for SCAPIS and 2016‐511‐31 for the linkage of register data to SCAPIS participants), and all participants provided written informed consent. The present analysis of de‐identified data was conducted under the same ethical framework and received additional approval (dnr 2024‐07175‐01).

## Supporting information




**Figure S1**: MASLD prevalence according to the cardiometabolic comorbidities (obesity, type 2 diabetes mellitus, hypertension, and hyperlipidemia) combinations.
**Table S1**: ICD codes for liver disease history assessment.
**Table S2**: MASLD prevalence in general population and advanced fibrosis prevalence in MASLD individuals stratified by the risk group (sex, BMI class, T2DM, hypertension, and hyperlipidemia).
**Table S3**: Univariable and multivariable Model 1 analyses of MASLD risk factors.
**Table S4**: Univariate risk factors for a high dAAR score suggestive of advanced fibrosis in participants with MASLD.

## Data Availability

The data used for this study were obtained from the SCAPIS under the petition number *PETITION‐691‐20250224*. The SCAPIS data are available based on the request of investigator(s) and the approval by the SCAPIS Program (www.scapis.org).
