# Supplementary material for: Prevalence and risk factors for metabolic dysfunction–associated steatotic liver disease in Sweden: Insights from the SCAPIS cohort
Source: J Intern Med. 2026 Feb 5;299(4):481–501. doi: 10.1111/joim.70071 (PMC12950636; doi:10.1111/joim.70071)
Supplement: Supplementary file 1 — Figure S1: MASLD prevalence according to the cardiometabolic comorbidities (obesity, type 2 diabetes mellitus, hypertension, and hyperlipidemia) combinations. Table S1: ICD codes for liver disease history assessment. Table S2: MASLD prevalence in general population and advanced fibrosis prevalence in MASLD individuals stratified by the risk group (sex, BMI class, T2DM, hypertension, and hyperlipidemia). Table S3: Univariable and multivariable Model 1 analyses of MASLD risk factors. Table S4: Univariate risk factors for a high dAAR score suggestive of advanced fibrosis in participants with MASLD. [file JOIM-299-481-s001.docx]

**Supplementary Appendix**

Supplementary Table S1. ICD codes for liver diseases history assessment

| Diagnosis | ICD-8 | ICD-9 | ICD-10 |
| --- | --- | --- | --- |
| Alcohol-related Liver Disease (ALD) | 571,00, 571,01 | 571.0-571.3 | K70 |
| Chronic viral hepatitis | 999,2, 070 | 070* | B16*, B17, B18,  B19 |
| Autoimmune liver disease:  Autoimmune Hepatitis (AIH) and Primary Biliary Cirrhosis (PBC) and Primary Sclerosing Cholangitis (PSC) | No available code | 571.6, 576.1 | K83.0A, K83.0F,  K74.3, K75.4 |
| Hemochromatosis | 273,2 | 275.0 | E83.1 |
| Wilson | 273,3 | 275.1 | E83.0B |
| Alpha-1-antitrypsin deficiency | No available code | 277.6 | E88.0A, E88.0B |
| Budd-Chiari | No available code | 453.0 | I82.0, K76.5 |
| Chronic hepatitis,  unspecified | 570 | 571.4 | K73.9, K73.2 |
| Secondary or unspecified biliary  cirrhosis | No available code | 571.6 | K74.4, K74.5 |
| Codes associated with alcohol use disorder | 303 | 303, 305.0 | F10 |
| Codes associated with somatic consequences of alcohol (except ALD) | 291, 980,00, 980,01,  980,99 | 291, 357.5, 425.5,  535.3, 980.1, 980.9 | E24.4, G62.1, I42.6, K29.2, G31.2,  G72.1, K85.2,  K86.0, T51.0,  T51.9, Y57.3, X65, Z50.2, Z71.4, Z72.1 |
| Codes associated with drug use disorders  except nicotine/caffeine | No available code | 305.1-9 | F11-F14, F16, F18, F19 |
| Cirrhosis, compensated | 571,9 | 571.5, 571.2  *(note, 571.2 is for specifically ALD- cirrhosis)* | K74.6,  K70.3, B18.1E/G,  B18.2E/G |
| Esophageal varices, not bleeding | No available code | 456.1, 456.21 | I85.9, I98.2 |

Supplementary Table S2: MASLD prevalence in general population and advanced fibrosis prevalence in MASLD individual stratified by the risk group (sex, BMI class, T2DM, hypertension, and hyperlipidemia)

|  | MASLD: HU<48 (liver fat>30%) | Advanced fibrosis in participants with MASLD (dAAR >1.5708) |
| --- | --- | --- |
| General population | 4933/27236 (18.1%) | 1225/4863 (25.2%) |
| Men | 3364/13170 (25.5%) | 889/3364 (26.43%) |
| Women | 1569/14066 (11.2%) | 336/1569 (21.4%) |
| BMI class |  |  |
| Normal weight | 275/9646 (2.9%) | 57/275 (20.7%) |
| Overweight | 1977/11544 (17.1%) | 485/1977 (24.5%) |
| Obese | 2661/5758 (46.2%) | 681/2661 (25.6%) |
| T2DM | 1209 /2653 (45.6%) | 338/1164 (29.0%) |
| Hypertension | 2742/9374 (29.3%) | 776/2742 (28.3%) |
| Hyperlipidemia | 2681/9592 (28.0%) | 722/2606 (27.7%) |
| Abbreviations: BMI, body mass index; T2DM, type 2 diabetes mellitus | |  |

Supplementary Table S3: Univariable and multivariable model 1 analyses of MASLD risk factors

| Univariate analyses | | | |  | Multivariable Model 1 | | | |
| --- | --- | --- | --- | --- | --- | --- | --- | --- |
|  | OR | 95% CI | p-value |  |  | OR | 95% CI | p-value |
| Age (years) | 1.04 | 1.03, 1.05 | <0.001 |  | **Age** | 1.04 | 1.03, 1.05 | <0.001 |
| Sex |  |  |  |  | **Sex** |  |  |  |
| Female | 1.0 | reference |  |  | Female | 1.0 | reference |  |
| Male | 2.73 | 2.56, 2.92 | <0.001 |  | Male | 2.19 | 2.01, 2.40 | <0.001 |
| Geographic origin |  |  |  |  | **Geographic origin** |  |  |  |
| Swedish | 1.0 | reference |  |  | Swedish | 1.0 | reference |  |
| Europe | 1.25 | 1.14, 1.37 | <0.001 |  | Europe | 0.82 | 0.72, 0.94 | 0.003 |
| North America | 1.15 | 0.60, 2.05 | 0.646 |  | North America | 1.02 | 0.45, 2.17 | 0.954 |
| South America | 1.54 | 1.15, 2.04 | 0.003 |  | South America | 1.08 | 0.73, 1.58 | 0.689 |
| Asia | 1.20 | 1.03, 1.40 | 0.019 |  | Asia | 0.72 | 0.58, 0.89 | 0.002 |
| Africa | 0.60 | 0.35, 0.97 | 0.048 |  | Africa | 0.52 | 0.26, 0.94 | 0.042 |
| Education level |  |  |  |  | **Education level** |  |  |  |
| Primary education | 1.0 | reference |  |  | Primary education | 1.0 | reference |  |
| Secondary education | 0.75 | 0.68, 0.83 | <0.001 |  | Secondary education | 1.03 | 0.90, 1.18 | 0.663 |
| Tertiary education | 0.46 | 0.42, 0.52 | <0.001 |  | Tertiary education | 0.97 | 0.84, 1.11 | 0.631 |
| Waist circumference | 1.13 | 1.12, 1.13 | <0.001 |  | **BMI class** |  |  |  |
| BMI (kg/m^2^) | 1.30 | 1.29, 1.31 | <0.001 |  | Non obesity | 1.0 | reference |  |
| BMI in class |  |  |  |  | Obesity | 13.4 | 11.6, 15.7 | <0.001 |
| Normal weight | 1.0 | reference |  |  | **T2DM, yes** | 1.88 | 1.72, 2.05 | <0.001 |
| Overweight | 7.04 | 6.20, 8.03 | <0.001 |  | **Hypertension, yes** | 1.45 | 1.34, 1.58 | <0.001 |
| Obese | 29.3 | 25.7, 33.4 | <0.001 |  | **ALT,** U/L | 1.05 | 1.05, 1.06 | <0.001 |
| T2DM, yes | 4.69 | 4.31, 5.10 | <0.001 |  | **AST,** U/L | 0.98 | 0.97, 0.99 | <0.001 |
| T2DM in class |  |  |  |  | **Exercise** |  |  |  |
| No T2DM | 1.0 | reference |  |  | Sedentary | 1.0 | reference |  |
| IFG | 2.45 | 2.25, 2.66 | <0.001 |  | Moderate exercise | 0.79 | 0.71, 0.89 | <0.001 |
| New T2DM | 6.96 | 5.98, 8.09 | <0.001 |  | Moderate but regular | 0.51 | 0.45, 0.59 | <0.001 |
| Treated T2DM | 6.51 | 5.70, 7.44 | <0.001 |  | Regular exercise | 0.37 | 0.31, 0.45 | <0.001 |
| Elevated HBA1c | 3.14 | 2.66, 3.71 | <0.001 |  | **Coffee consumption** |  |  |  |
| Hypertension, yes | 2.96 | 2.78, 3.15 | <0.001 |  | No or less than 1 time a week | 1.0 | reference |  |
| Hypertension class |  |  |  |  | Once a day | 1.72 | 1.20, 2.44 | 0.003 |
| No HBP | 1.0 | reference |  |  | 2-3 times a day | 1.32 | 1.16, 1.51 | <0.001 |
| Prehypertension | 2.24 | 2.03, 2.47 | <0.001 |  | 4 times a day or more often | 1.70 | 1.37, 2.10 | <0.001 |
| HBP not treated | 3.09 | 2.83, 3.37 | <0.001 |  | **Soda intake** |  |  |  |
| HBP treated and controlled | 3.92 | 3.60, 4.26 | <0.001 |  | No or less than times per week | 1.0 | reference |  |
| HBP treated but uncontrolled | 4.89 | 4.21, 5.67 | <0.001 |  | Once a day | 1.11 | 0.99, 1.24 | 0.087 |
| Hyperlipidemia, yes | 2.66 | 2.49, 2.83 | <0.001 |  | 2 times a day or more often | 1.40 | 1.11, 1.76 | 0.004 |
| Hyperlipidemia in class |  |  |  |  | **Smoking status** |  |  |  |
| Not hyperlipidemia | 1.0 | reference |  |  | Never | 1.0 | reference |  |
| Hyperlipidemia not treated | 2.57 | 2.40, 2.75 | <0.001 |  | Former smoker | 1.22 | 1.12, 1.33 | <0.001 |
| Treated Hyperlipidemia | 2.98 | 2.69, 3.31 | <0.001 |  | Current | 1.32 | 1.16, 1.50 | <0.001 |
| Comorbidities combination without hyperlipidemia |  |  |  |  | **Alcohol consumption, g/d** | 1.04 | 1.03, 1.04 | <0.001 |
| No comorbidities | 1.0 | reference |  |  |  |  |  |  |
| T2DM alone | 3.75 | 3.11, 4.50 | <0.001 |  |  |  |  |  |
| HBP alone | 2.37 | 2.15, 2.62 | <0.001 |  |  |  |  |  |
| Obesity alone | 7.00 | 6.29, 7.79 | <0.001 |  |  |  |  |  |
| T2DM and HBP | 7.32 | 6.21, 8.62 | <0.001 |  |  |  |  |  |
| Obesity and T2DM | 20.9 | 16.8, 26.1 | <0.001 |  |  |  |  |  |
| Obesity and HBP | 11.9 | 10.7, 13.2 | <0.001 |  |  |  |  |  |
| Obesity, T2DM, and HBP | 31.3 | 26.5, 37.0 | <0.001 |  |  |  |  |  |
| Comorbidities combination with hyperlipidemia |  |  |  |  |  |  |  |  |
| No comorbidities | 1.0 | reference |  |  |  |  |  |  |
| HBP alone | 2.52 | 2.20, 2.89 | <0.001 |  |  |  |  |  |
| Hyperlipidemia alone | 2.56 | 2.25, 2.92 | <0.001 |  |  |  |  |  |
| T2DM alone | 3.39 | 2.56, 4.42 | <0.001 |  |  |  |  |  |
| HBP and hyperlipidemia | 4.72 | 4.11, 5.41 | <0.001 |  |  |  |  |  |
| T2DM and HBP | 7.60 | 5.71, 10.0 | <0.001 |  |  |  |  |  |
| T2DM and hyperlipidemia | 8.53 | 6.57, 11.0 | <0.001 |  |  |  |  |  |
| Obesity alone | 7.80 | 6.74, 9.02 | <0.001 |  |  |  |  |  |
| T2DM, HBP, and hyperlipidemia | 11.9 | 9.68, 14.6 | <0.001 |  |  |  |  |  |
| Obesity and HBP | 13.7 | 11.8, 15.8 | <0.001 |  |  |  |  |  |
| Obesity and hyperlipidemia | 13.1 | 11.2, 15.3 | <0.001 |  |  |  |  |  |
| Obesity and T2DM | 18.5 | 13.5, 25.3 | <0.001 |  |  |  |  |  |
| Obesity, HBP, and hyperlipidemia | 20.1 | 17.3, 23.2 | <0.001 |  |  |  |  |  |
| Obesity, T2DM, and hyperlipidemia | 45.6 | 33.2, 63.4 | <0.001 |  |  |  |  |  |
| Obesity, T2DM, HBP, and hyperlipidemia | 45.8 | 37.4, 56.3 | <0.001 |  |  |  |  |  |
| ALT, U/L | 1.06 | 1.05, 1.07 | <0.001 |  |  |  |  |  |
| AST, U/L | 1.05 | 1.04, 1.06 | <0.001 |  |  |  |  |  |
| GGT, IU/L | 1.02 | 1.01, 1.03 | <0.001 |  |  |  |  |  |
| TGs ≥1.7 mmol/L | 2.77 | 2.65, 2.89 | <0.001 |  |  |  |  |  |
| Total cholesterol >5.5 mmol/L | 0.89 | 0.86, 0.91 | <0.001 |  |  |  |  |  |
| HDL-C, <1.0 mmol/L for men (<1.3 mmol/L for women) | 0.12 | 0.11, 0.13 | <0.001 |  |  |  |  |  |
| Fasting glucose >6.9 mmol/L | 1.99 | 1.92, 2.07 | <0.001 |  |  |  |  |  |
| HbA1c >6.4% | 1.09 | 1.08, 1.10 | <0.001 |  |  |  |  |  |
| Exercise |  |  |  |  |  |  |  |  |
| Sedentary | 1.0 | reference |  |  |  |  |  |  |
| Moderate but not regular | 0.53 | 0.48, 0.58 | <0.001 |  |  |  |  |  |
| Moderate but regular | 0.26 | 0.23, 0.29 | <0.001 |  |  |  |  |  |
| Regular exercise | 0.17 | 0.15, 0.20 | <0.001 |  |  |  |  |  |
| Coffee consumption |  |  |  |  |  |  |  |  |
| No or less 1 time a week | 1.0 | reference |  |  |  |  |  |  |
| Once a day | 2.03 | 1.55, 2.63 | <0.001 |  |  |  |  |  |
| 2-3 times a day | 1.24 | 1.12, 1.37 | <0.001 |  |  |  |  |  |
| 4 times a day or more often | 1.64 | 1.40, 1.91 | <0.001 |  |  |  |  |  |
| Soda intake |  |  |  |  |  |  |  |  |
| No or less than times per week | 1.0 | reference |  |  |  |  |  |  |
| Once a day | 0.95 | 0.86, 1.03 | 0.214 |  |  |  |  |  |
| 2 times a day or more often | 1.54 | 1.29, 1.82 | <0.001 |  |  |  |  |  |
| Smoking status |  |  |  |  |  |  |  |  |
| Never | 1.0 | reference |  |  |  |  |  |  |
| Former smoker | 1.41 | 1.32, 1.51 | <0.001 |  |  |  |  |  |
| Current smoker | 1.48 | 1.34, 1.63 | <0.001 |  |  |  |  |  |
| Alcohol consumption, g/d | 1.04 | 1.03, 1.04 | <0.001 |  |  |  |  |  |
| Abbreviations: OR, Odds ratios; CI, Confidential intervales; BMI, body mass index; T2DM, type 2 diabetes mellitus; IFG, impaired fasting glucose; HbA1c, hemoglobin [A1C](https://medlineplus.gov/a1c.html); HBP, high blood pressure (hypertension); ALT, alanine transaminase. AST: aspartate aminotransferase; GGT, gamma-glutamyl transferase; TGs, triglycerides; HDL-C, high-density lipoprotein cholesterol; g/d, gram/day.  Multivariable Model 1: BMI class (obese vs non obese); T2DM (Yes vs No), Hypertension (Yes vs No); Hyperlipidemia (Yes vs No) and adjusted for age, sex, geographic origin, education level, AST, ALT, GGT, Sprot practice, coffee consumption, soda intake, smoking, and alcohol consumption | | | | | | | | |

Supplementary Table S4: Univariate risk factors for a high dAAR score suggestive of advanced fibrosis in participants with MASLD

|  | Univariate analyses | | |
| --- | --- | --- | --- |
|  | OR | 95% CI | p-value |
| Sex |  |  |  |
| Women | 1.0 | reference |  |
| Men | 1.28 | 1.11, 1.48 | <0.001 |
| Geographic origin |  |  |  |
| Swedish | 1.0 | reference |  |
| Europe | 0.82 | 0.67, 0.99 | 0.040 |
| North America | 2.00 | 0.59, 6.28 | 0.238 |
| South America | 1.00 | 0.54, 1.73 | 0.987 |
| Asia | 0.75 | 0.53, 1.05 | 0.099 |
| Africa | 0.60 | 0.14, 1.84 | 0.422 |
| Education level |  |  |  |
| Primary education | 1.0 | reference |  |
| Secondary education | 1.05 | 0.86, 1.30 | 0.634 |
| Tertiary education | 1.07 | 0.87, 1.34 | 0.521 |
| Waist circumference | 1.01 | 1.01, 1.02 | <0.001 |
| BMI, (kg/m^2^) | 1.01 | 1.00, 1.02 | 0.179 |
| BMI in class |  |  |  |
| Normal weight | 1.0 | reference |  |
| Overweight | 1.27 | 0.94, 1.74 | 0.13 |
| Obese | 1.35 | 1.00, 1.84 | 0.055 |
| T2DM, yes | 1.26 | 1.09, 1.46 | 0.002 |
| T2DM in class |  |  |  |
| No T2DM | 1.0 | reference |  |
| IFG | 1.27 | 1.07, 1.50 | 0.006 |
| New diagnosed T2DM | 1.89 | 1.49, 2.38 | <0.001 |
| Treated T2DM | 1.23 | 0.98, 1.54 | 0.069 |
| Elevated HBA1c | 1.01 | 0.72, 1.39 | 0.890 |
| Hypertension, yes | 1.53 | 1.34, 1.75 | <0.001 |
| Hypertension class |  |  |  |
| No hypertension | 1.0 | reference |  |
| Prehypertension | 1.40 | 1.13, 1.74 | 0.002 |
| Hypertension, not treated | 1.52 | 1.26, 1.84 | <0.001 |
| Hypertension treated and controlled | 1.83 | 1.53, 2.18 | <0.001 |
| Hypertension treat but uncontrolled | 2.00 | 1.52, 2.64 | <0.001 |
| Hyperlipidemia, yes | 1.29 | 1.13, 1.47 | <0.001 |
| Hyperlipidemia in class |  |  |  |
| Not hyperlipidemia | 1.0 | reference |  |
| Hyperlipidemia not treated | 1.21 | 1.05, 1.40 | 0.007 |
| Hyperlipidemia treated | 1.55 | 1.27, 1.89 | <0.001 |
| Comorbidities combination without hyperlipidemia |  |  |  |
| No comorbidities | 1.0 | reference |  |
| T2DM alone | 1.29 | 0.95, 1.75 | 0.100 |
| Hypertension alone | 1.61 | 1.25, 2.06 | <0.001 |
| Obesity alone | 0.93 | 0.71, 1.22 | 0.589 |
| T2DM and hypertension | 1.61 | 1.22, 2.12 | <0.001 |
| Obesity and T2DM | 1.29 | 0.96, 1.74 | 0.091 |
| Obesity and hypertension | 1.44 | 1.13, 1.84 | 0.003 |
| Obesity, T2DM, and hypertension | 1.93 | 1.54, 2.43 | <0.001 |
| Comorbidities combination with hyperlipidemia |  |  |  |
| No comorbidities | 1.0 | reference |  |
| Hypertension alone | 1.58 | 1.09, 2.30 | 0.017 |
| Hyperlipidemia alone | 1.47 | 1.03, 2.10 | 0.033 |
| T2DM alone | 1.47 | 0.94, 2.26 | 0.087 |
| Hypertension and hyperlipidemia | 2.33 | 1.64, 3.33 | <0.001 |
| T2DM and hypertension | 2.32 | 1.53, 3.52 | <0.001 |
| T2DM and hyperlipidemia | 1.72 | 1.09, 2.67 | 0.017 |
| Obesity alone | 0.83 | 0.53, 1.26 | 0.384 |
| T2DM, hypertension, and hyperlipidemia | 1.76 | 1.19, 2.58 | 0.004 |
| Obesity and hypertension | 1.39 | 0.96, 2.02 | 0.085 |
| Obesity and hyperlipidemia | 1.37 | 0.94, 2.00 | 0.101 |
| Obesity and T2DM | 1.41 | 0.91, 2.18 | 0.119 |
| Obesity, hypertension, and hyperlipidemia | 2.11 | 1.50, 2.98 | <0.001 |
| Obesity, T2DM, and hyperlipidemia | 1.75 | 1.15, 2.67 | 0.009 |
| Obesity, T2DM, hypertension, and hyperlipidemia | 2.27 | 1.65, 3.14 | <0.001 |
| TGs ≥1.7 mmol/L | 1.11 | 1.05, 1.18 | <0.001 |
| Total cholesterol >5.5 mmol/L | 0.99 | 0.94, 1.05 | 0.762 |
| HDL-C <1.0 mmol/L for men (<1.3 mmol/L for women) | 0.96 | 0.81, 1.14 | 0.652 |
| Fasting glucose >6.9 mmol/L | 1.08 | 1.04, 1.12 | <0.001 |
| Exercise |  |  |  |
| Sedentary | 1.0 | reference |  |
| Moderate but not regular | 1.09 | 0.92, 1.29 | 0.335 |
| Moderate but regular | 1.01 | 0.82, 1.25 | 0.906 |
| Regular exercise | 0.88 | 0.62, 1.23 | 0.464 |
| Coffee consumption |  |  |  |
| No or less 1 time a week | 1.0 | reference |  |
| Once a day | 1.16 | 0.69, 1.88 | 0.553 |
| 2-3 times a day | 1.22 | 1.00, 1.49 | 0.048 |
| 4 times a day or more often | 1.19 | 0.87, 1.60 | 0.275 |
| Soda intake |  |  |  |
| No or less than times per week | 1.0 | reference |  |
| Once a day | 1.05 | 0.87, 1.26 | 0.596 |
| 2 times a day or more often | 0.91 | 0.64, 1.29 | 0.596 |
| Smoking status |  |  |  |
| Never | 1.0 | reference |  |
| Former smoker | 1.02 | 0.88, 1.17 | 0.790 |
| Current smoker | 0.91 | 0.74, 1.11 | 0.353 |
| Alcohol consumption, g/day | 1.03 | 1.02, 1.04 | <0.001 |
| Abbreviations: OR, odds ratios; CI, confidential intervals; BMI, body mass index; T2DM, type 2 diabetes mellitus; IFG, impaired fasting glucose; HbA1c, hemoglobin [A1C](https://medlineplus.gov/a1c.html); TGs, triglycerides; HDL-C, high-density lipoprotein cholesterol | | | |


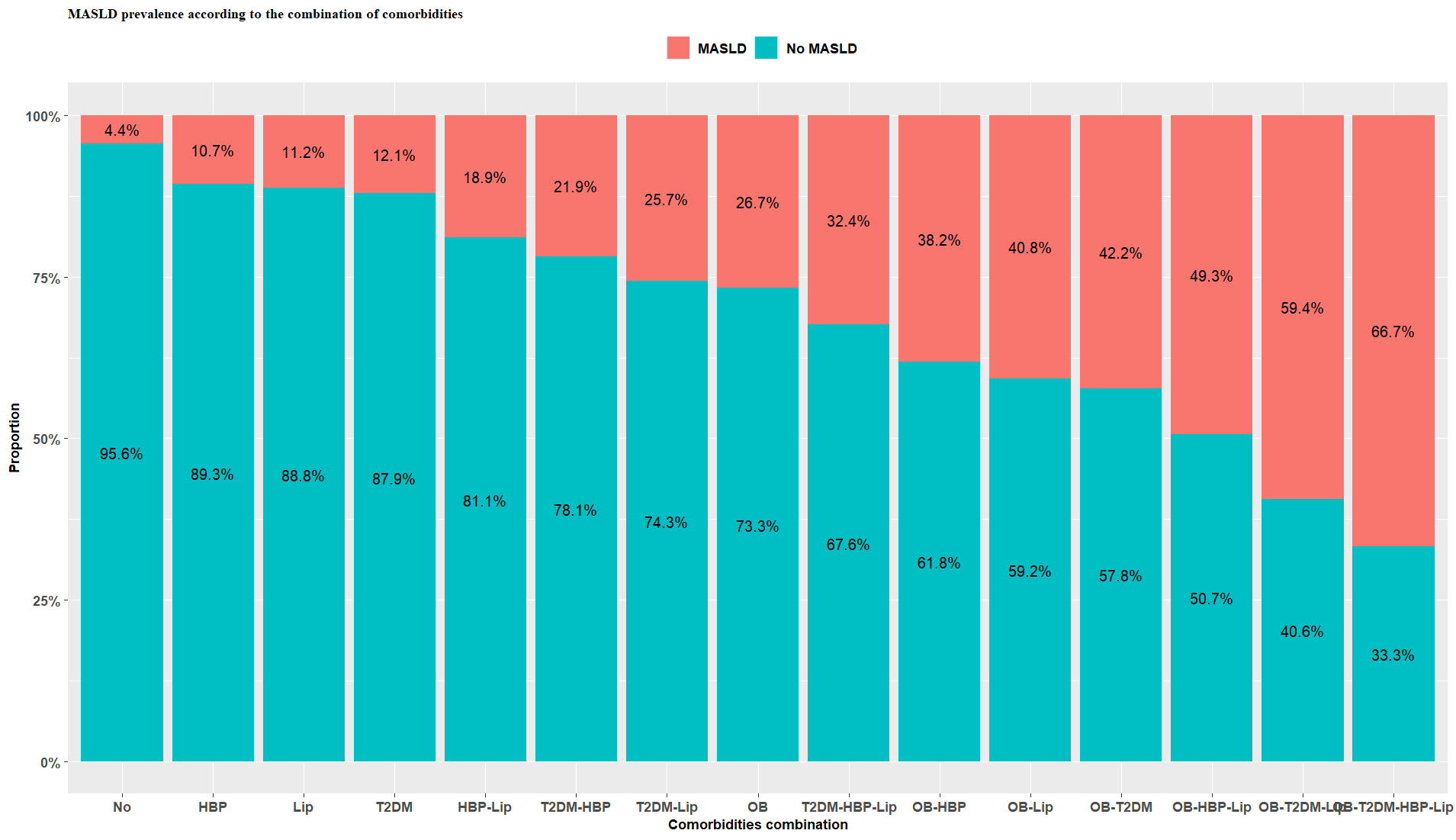


Abbreviations: No: non comorbidity; HBP, hypertension; Lip, hyperlipidemia; T2DM, type 2 diabetes mellitus; OB, obesity

Figure S1: MASLD prevalence according to the cardiometabolic comorbidities (obesity, type 2 diabetes mellitus, hypertension, and hyperlipidemia) combinations.
